# Labeling and Localization Strategies for In Situ Cryo-Electron Tomography Across the Viral Life Cycle

**DOI:** 10.3390/v18070790

**Published:** 2026-07-19

**Authors:** Yoon Ho Park, Rana Kim, Kun-Ho Song, Hyun Suk Jung

**Affiliations:** 1 Department of Biochemistry, College of Natural Sciences, Kangwon National University, Chuncheon 24341, Republic of Korea; 2 University-Industry Cooperation Foundation, Kangwon National University, Chuncheon 24341, Republic of Korea

**Keywords:** Cryo-electron tomography, in situ structural virology, labeling strategy, Cryo-CLEM, viral replication organelle

## Abstract

Cryo-electron tomography (Cryo-ET) has emerged as a transformative tool for visualizing viral components within their native cellular environment, enabling structural interrogation of viral life cycle events at nanometer resolution without chemical fixation or heavy metal staining. However, a persistent challenge in applying Cryo-ET to virus research is the unambiguous identification of specific viral components within densely crowded tomographic volumes. Electron density encodes mass and shape but not molecular identity, and as the cellular environment grows more complex, the assumption that a given density has no plausible alternative assignment becomes increasingly difficult to defend. This review surveys labeling and localization strategies for in situ Cryo-ET of viral components, encompassing label-free exploitation of native electron density, Cryo-immunogold labeling, genetically encoded and synthetic molecular tags, and correlative Cryo-light/electron microscopy (Cryo-CLEM) combined with Cryo-focused ion beam (Cryo-FIB) milling. We first summarize the landmark structural discoveries that in situ Cryo-ET has delivered across virus families, and then evaluate each labeling strategy against the structural and functional constraints that viral proteins impose, providing a practical framework for matching a labeling approach to a specific viral component and life-cycle stage.

## 1. Introduction

The molecular events of the viral life cycle unfold within the complex three-dimensional architecture of the host cell. Understanding these events at the structural level requires imaging methods that preserve the native cellular context while achieving sufficient resolution to resolve macromolecular assemblies. Cryo-electron tomography (Cryo-ET) fulfills this requirement uniquely. By rapid vitrification, biological specimens are immobilized in near-native conformational states, and when combined with focused ion beam (FIB) milling and subtomogram averaging, Cryo-ET enables in situ structure determination at resolutions approaching 3–4 Å for ordered complexes [1,2]. Landmark demonstrations include the 3.9 Å structure of immature HIV-1 capsid-SP1 within intact virus particles [3] and the 3.5 Å in-cell ribosome structure achieved with multi-particle refinement [4]. These advances rest on a broad ecosystem of GPU-accelerated subtomogram averaging software, including RELION [5], Warp and M [4,6], Dynamo [7], and emClarity [8], whose complementary implementations have collectively pushed these resolution boundaries.

Yet a persistent challenge is the problem of molecular identification. How does one unambiguously assign a density observed in a tomogram to a specific viral component, particularly when that component lacks a distinctive morphological signature or co-localizes with structurally similar host proteins? Viral systems possess a distinctive labeling context that sets them apart from studies of general cellular proteins. Many viral assemblies carry highly repetitive protein lattices or precisely defined geometries that generate recognizable electron density patterns, making label-free identification feasible in a wider range of scenarios than for monomeric host proteins. At the same time, viral proteins are often exquisitely sensitive to structural perturbation, and fusion of even moderately sized tags can disrupt capsid assembly stoichiometry or impair polymerase processivity [9]. Furthermore, the relevant viral components change at each life-cycle stage, so labeling strategies must be matched not only to the protein but also to the specific cellular context and kinetic window of interest.

The contrast between early chemically fixed electron microscope tomography (EMT) and modern Cryo-ET illustrates why labeling strategy matters. Kopek et al. [10] provided the first three-dimensional model of a Flock House virus (FHV) replication organelle using conventional EMT with chemical fixation and heavy metal staining, successfully localizing replicase and nascent RNA by immunogold. However, the spherule neck crown complex, a 12-fold symmetric structure later revealed by Cryo-ET [11], was completely undetectable in the chemically fixed preparations, illustrating the critical gain in structural fidelity afforded by vitrification and the corresponding need for labeling approaches compatible with Cryo-preservation.

This review surveys labeling and localization strategies for in situ Cryo-ET of viral components across the viral life cycle. The strategies are presented not in the chronological order in which they were developed, but along a continuum of invasiveness and information content. Label-free imaging perturbs nothing but confers no molecular identity. Immunogold labeling, inherited from classical electron microscopy, confers identity but demands physical access to the epitope. Genetic and synthetic tags seek to supply identity inside the cell, at the cost of a covalent modification of the target. Cryo-CLEM supplies identity indirectly, through fluorescence, when the event of interest is rare. Landmark structural discoveries delivered by in situ Cryo-ET across virus families, together with the labeling strategy each required, are summarized in Table 1. Three case studies then ground the discussion in concrete biological outcomes. FHV replication organelles in Drosophila S2 cells demonstrate complementary label-free and immunogold approaches. HIV-1 nuclear import in permeabilized T cells exemplifies the full Cryo-CLEM and Cryo-FIB-ET workflow. The Copia long terminal repeat (LTR) retrotransposon in intact Drosophila egg chambers extends the methodology to multicellular tissue via Cryo-FIB lift-out. These three systems were selected because they jointly span the three labeling regimes distinguished in this review (label-free sufficiency, immunogold-assisted confirmation, and obligatory correlative targeting), because they span three classes of specimen (a cultured cell line, a permeabilized cell, and an intact multicellular tissue), and because they sample three distinct stages of the viral life cycle: genome replication, nuclear import, and particle assembly. Together they provide a practical decision framework for labeling strategy selection.

## 2. Label-Free Cryo-ET: When Morphology Is the Label

In label-free Cryo-ET, viral components are identified solely on the basis of their intrinsic electron scattering properties, morphological features, and spatial context [1]. Icosahedral capsids and helical nucleocapsids present highly ordered, repetitive protein lattices with predictable local symmetry, enabling template-matching algorithms to locate and extract subtomogram particles with high sensitivity [8]. Dense RNA packing within replication compartments generates high electron density relative to the surrounding cytoplasm. Viral envelope glycoproteins form defined trimeric or dimeric complexes at the virion surface that can be discriminated from host membrane proteins by size, shape, and spacing. For a growing number of viruses, these features enable structural interpretation without any labeling step, preserving the sample in its most native state [27].

The most comprehensively characterized example is the FHV RNA replication compartment. Cryo-ET of isolated mitochondria from FHV-infected Drosophila S2 cells revealed the interior of the replication spherule—electron-dense fibrillar material interpreted as double-stranded RNA (dsRNA)—and, gating its neck, a 12-fold symmetric crown complex, both features completely obscured in earlier chemically fixed preparations [10,11]. Subtomogram averaging subsequently resolved this crown to subnanometer resolution (8.5 Å), yielding the first detailed structural view of a (+)RNA virus replication complex crown [12] and building on decades of work on (+)RNA virus replication organelles [28,29]. The same logic applies wherever a viral assembly is both ordered and physically enclosed. Coronavirus genome replication proceeds inside a sealed double-membrane vesicle (DMV), and in situ Cryo-ET revealed the approximately 3 MDa nsp3–nsp4 pore complex that spans it, the conduit through which nascent viral RNA reaches the cytosol [13,14], building on comprehensive in situ characterization of SARS-CoV-2 replication [30]. That this architecture became visible at all is a consequence of the labeling problem it solves: the pore lies within the DMV lumen, inaccessible to any antibody without membrane disruption, so label-free imaging was not merely convenient but the only route to it. Analogous neck complexes have been described for chikungunya virus (CHIKV) spherules at the plasma membrane [17], in which the nsP1 dodecameric ring shares a comparable 12-fold topology with the FHV crown yet is mechanistically and structurally distinct [18], illustrating that convergent symmetry does not imply functional or evolutionary equivalence.

Label-free Cryo-ET extends beyond replication organelles to virion structure, trafficking, and assembly. Subtomogram averaging of HIV-1 Env trimers [31], the conformational states and hinge-mediated flexibility of the SARS-CoV-2 spike on intact virions [15,16], progressive nucleocapsid condensation intermediates in Ebola virus-infected cells [19,20], endolysosomal trafficking of human papillomavirus type 16 (HPV16), which in a recent preprint has been proposed to challenge the prevailing lysosomal disassembly model [22], clustering of influenza A virus viral ribonucleoproteins (vRNPs) at Rab11a-positive recycling endosomes [21], and the mature HIV-1 capsid both within intact virions [23] and in transit through an intact nuclear pore complex (NPC) [24] were all characterized without any exogenous label. These and other landmark discoveries are summarized in Table 1.

The same principles are being applied well beyond the systems emphasized in this review. Electron tomography has resolved the membrane architecture and biogenesis of picornavirus replication organelles [32], and comparable membrane morphotypes recur across flaviviruses and hepaciviruses [33], while herpesvirus capsids have been resolved in situ within cell nuclei by correlative light and Cryo-ET [34]. The generalization is that label-free identification succeeds in proportion to the order, size, and abundance of the target, and not in proportion to the taxonomic familiarity of the virus. The strategies discussed here are therefore expected to transfer to viral families not explicitly treated below.

The label-free approach fails precisely where viral biology is most complex. Monomeric viral proteins that lack repetitive symmetry, such as most non-structural accessory proteins, are unidentifiable by template matching or visual inspection [1,27]. Viral RNA cannot be distinguished from host RNA by electron density alone, meaning that within a replication organelle one cannot determine whether a given density represents positive-strand genome, negative-strand intermediate, or subgenomic RNA. Partially assembled capsids, budding intermediates, and disassembly fragments deviate from mature-particle templates and are frequently missed or misassigned, a failure mode that motivated the development of statistically calibrated template-matching frameworks reporting explicit false-discovery rates [35]. Most critically, in densely infected cells where the cytoplasm is crowded with replication organelles, viral particles, and host stress responses, morphologically similar structures from virus and host can overlap, and without molecular identity confirmation the assignment remains presumptive. This is not a technical limitation that better detectors or algorithms will solve. It is a fundamental information deficit that arises because electron density encodes mass and shape but not molecular identity. Every label-free structural claim in Cryo-ET therefore carries an implicit assumption that the observed density has no plausible alternative assignment, an assumption that becomes increasingly difficult to defend as the cellular environment grows more complex [35].

This fundamental identification deficit is compounded by technical constraints inherent to Cryo-ET data acquisition. Sample thickness directly determines tilt-series quality, as electrons traversing thicker specimens undergo increased multiple scattering that degrades contrast and complicates contrast transfer function (CTF) correction. Cryo-FIB milling has become essential for preparing electron-transparent lamellae from thick cellular specimens [36,37]. Cumulative electron dose of approximately 60–120 e^−^/Å^2^ must be distributed across 40–60 tilt images, with dose-symmetric tilt-schemes optimized for high-resolution sub-tomogram averaging [38], leaving each individual image at extremely low signal-to-noise ratios that may be insufficient for detecting viral components lacking high symmetry or large copy numbers. Detectability in a raw tomographic volume is therefore set by the specific combination of target abundance, symmetry order, and specimen thickness. It does not follow from the morphological distinctiveness of a target in a published subtomogram average, which reflects the coherent averaging of many copies.

## 3. Immunogold Labeling: Molecular Identity at the Cost of Access

Immunogold electron microscopy, in which a primary antibody is detected by a secondary antibody conjugated to an electron-dense gold nanoparticle (typically 5, 10, or 15 nm), has long been used in virology for localizing specific proteins within cellular ultrastructure. Historically, antibody–gold conjugates entered electron microscopy decades before direct electron detectors and template-matching algorithms made label-free identification of viral assemblies practical, and immunogold is therefore the inherited starting point from which Cryo-ET labeling strategies subsequently diverged. In contemporary Cryo-ET, however, its application remains comparatively uncommon, for the reasons developed in this section. A representative bridge between classical and cryogenic immunogold is the vitrification of Tokuyasu-style immuno-labeled sections for correlative cryo-light and electron microscopy [39]. The foundational application to viral replication compartments was established by Kopek et al. [10], who demonstrated that 88 ± 5% of anti-protein A immunogold signal localized to mitochondrial spherules in FHV-infected Drosophila S2 cells, with a parallel anti-BrU (5-bromouridine) experiment confirming spherules as sites of active RNA synthesis. These experiments provided the first direct functional link between spherule morphology and replicase localization. However, the chemical fixation and resin embedding required for classical immunogold necessarily introduce artifacts including membrane deformation and loss of macromolecular contrast, motivating the development of Cryo-compatible alternatives.

Native Cryo-immunogold labeling eliminates chemical fixation by performing antibody-gold labeling on unfixed specimens adsorbed to Cryo-EM grids under near-physiological conditions prior to plunge-freezing. Yi et al. [40] demonstrated this on HIV-1-infected cells, localizing the host restriction factor tetherin (BST-2) with 6 nm gold particles while preserving native membrane morphology. The same study applied the approach to respiratory syncytial virus (RSV) F glycoprotein on intact viral particles, confirming that antigenic epitopes of surface glycoproteins remain accessible for immunogold labeling under native Cryo conditions. In the FHV system, Ertel et al. [11] used native Cryo-immunogold with anti-protein A antiserum to confirm that the viral replicase is a constituent of the crown complex, providing direct molecular identity to a feature defined purely by electron density in label-free tomograms.

The fundamental problem with immunogold in viral Cryo-ET is that most viral proteins of interest are physically inaccessible to antibodies. Replicases operate inside membrane-enclosed replication organelles. The FHV protein A crown sits at the spherule neck with its catalytic domains facing the spherule lumen, and the Ertel et al. labeling succeeded only because isolated mitochondria expose the cytoplasmic face of the crown where accessible epitopes happen to reside. For the SARS-CoV-2 DMV pore, the nsp3/nsp4 complex is entirely enclosed within the double membrane [13,14], and no amount of antibody optimization can overcome the physical barrier of two sealed lipid bilayers without destroying the structure one wishes to image. Envelope glycoproteins are the exception rather than the rule, as they are the only viral proteins routinely surface-exposed and antibody-accessible without permeabilization.

Beyond accessibility, the spatial uncertainty of 25–30 nm introduced by the primary antibody plus gold-conjugated secondary antibody is a serious constraint for viral complexes. The FHV crown’s central turret is ~19 nm in diameter, and its twelve subunits are spaced only a few nanometres apart [11,12]. A 25–30 nm localization uncertainty means that immunogold cannot assign protein A to a specific subunit position within the crown, only to the crown as a whole. For smaller viral complexes or for distinguishing between closely spaced viral and host proteins at membrane interfaces, this resolution is insufficient.

Whether a given antibody remains reactive under native Cryo conditions cannot be predicted in advance. The anti-protein A antiserum used by Ertel et al. retained antigenicity in vitrified specimens, but antigenicity is established empirically for each antibody–antigen pair rather than inferred from performance in fixed or denaturing formats. Immunogold in Cryo-ET therefore requires antibodies raised against native, conformational epitopes, and establishing that any given antibody is specific for immuno-electron microscopy is itself a demanding exercise [41]. For viruses lacking well-characterized antibody panels, particularly emerging or neglected pathogens, the screening effort required to identify such an antibody may be prohibitive.

A further constraint, inherited from classical immunogold electron microscopy, is incomplete labeling efficiency. Only a fraction of antigen-bound antibodies carries a detectable gold particle, so the density of gold observed in a tomogram systematically underestimates the density of the target. Monovalent Fab–Nanogold conjugates (1.4 nm) reduce both the linkage error and the steric burden and improve the fraction of labeled epitopes [42], but detection remains substoichiometric, and labeling efficiency is rarely quantified in published Cryo-ET studies [41]. The practical consequence is that immunogold supports the assignment of a molecular identity to a density that has already been observed, but does not support quantitative statements about the abundance or occupancy of the labeled protein.

## 4. Genetic and Synthetic Tags: Promise Constrained by Viral Assembly Logic

For Cryo-ET, a viable genetic tag must generate sufficient electron density contrast, present a recognizable structural morphology, and minimally perturb the fusion partner. Silvester and Baker [43] catalogued available strategies for general cellular applications. Here, we focus on why these tags face uniquely severe constraints when applied to viral proteins.

Ferritin assembles into a 10–12 nm cage that generates strong electron density when loaded with iron. The original ferritin-based label for cellular Cryo-ET was pioneered by Wang et al. [44]. The inducible FerriTag, which exploits rapamycin-induced FK506-binding protein (FKBP)/FKBP–rapamycin-binding (FRB) domain dimerization to recruit ferritin to a tagged target protein, was introduced by Clarke and Royle [45] and has since been adapted for Cryo-ET [46], offering temporal control over labeling. In the viral context, the rapamycin requirement introduces a problem beyond simple pharmacological side effects. Many viruses actively manipulate the mechanistic target of rapamycin (mTOR) pathway to facilitate replication organelle biogenesis, membrane remodeling, and translational control [47]. Rapamycin-mediated mTOR inhibition can therefore alter the very viral processes one intends to image, creating a confound that is not merely a background variable but a direct perturbation of the biological question.

The genetically encoded multimeric (GEM) tag system, developed by Fung et al. [48] and based on engineered encapsulin particles, self-assembles into ~25 nm icosahedral cages with a structural signature detectable by neural network classifiers. The size constraint is the central problem for viral applications. Capsid and matrix proteins polymerize into lattices whose subunit interfaces are separated by only a few nanometres; in the influenza M1 endoskeleton, for example, adjacent polymer strands are spaced approximately 3.6 nm apart [49], and the HIV-1 CA hexamer is built from subunit contacts on the same scale [50,51]. The relevant evidence that even much smaller tags perturb such lattices comes from fluorescent protein fusions, which are an order of magnitude smaller than any electron-dense cage. A ~3 nm EGFP inserted into HIV-1 Gag yields particles of reduced infectivity, and wild-type morphology and infectivity are recovered only when Gag-EGFP is co-expressed with untagged Gag so that the tagged subunit is diluted within the lattice [52]; comparable constraints govern the placement of fluorescent tags on other HIV-1 structural and enzymatic proteins [9]. If a 3 nm tag must be diluted to preserve lattice assembly, a 25 nm cage occupying a volume some three orders of magnitude larger cannot plausibly be accommodated at any capsid subunit interface. We therefore regard the incompatibility as a prediction from steric first principles rather than an experimentally demonstrated result: to our knowledge no attempt to fuse a GEM particle to a viral capsid protein has been reported, and correspondingly no counterexample exists. On this reasoning, GEM applicability in virology is restricted to replicase or accessory proteins that function as monomers or small oligomers and do not participate in ordered assembly.

DNA origami-based tags such as the signposts, referred to as SPOT, developed by Silvester et al. [53] create markers with uniquely identifiable shapes through programmable DNA geometry. Their 30–100 nm footprints and requirement for DNA-conjugated antibodies for delivery restrict them to extracellular surface proteins. This means SPOT can in principle label envelope glycoproteins on intact virions but cannot access replicases, capsid proteins during intracellular assembly, or viral RNA. For the majority of viral life- cycle events that occur inside the cell, DNA origami tags are inapplicable by design.

Two classes of label that are widely used in room-temperature cellular electron microscopy are, for principled reasons, unavailable in Cryo-ET. Peroxidase-based genetic tags such as APEX2 generate contrast indirectly, by polymerizing diaminobenzidine into an osmiophilic precipitate that must subsequently be stained with osmium tetroxide [54]; both the polymerization chemistry and the heavy-metal stain are incompatible with vitrification, which is defined by the absence of chemical fixation and stain. Combining APEX2 with high-pressure freezing and freeze substitution restores ultrastructural preservation, but reintroduces dehydration and staining, and therefore yields a room-temperature electron tomogram rather than a Cryo-ET volume. Metal-binding tags such as metallothionein avoid exogenous stain by chelating heavy-metal ions in situ [55], but the resulting cluster contributes little scattering contrast in a low-dose tomogram, metal loading is difficult to control in living cells, and the approach has failed for some multiprotein assemblies even under favorable room-temperature conditions [56]. Ferritin, GEM, and DNA origami remain the only tag classes that generate intrinsic Cryo-ET contrast without violating the constraints of vitrification, which is why the discussion in this section is confined to them.

**Figure 1:**
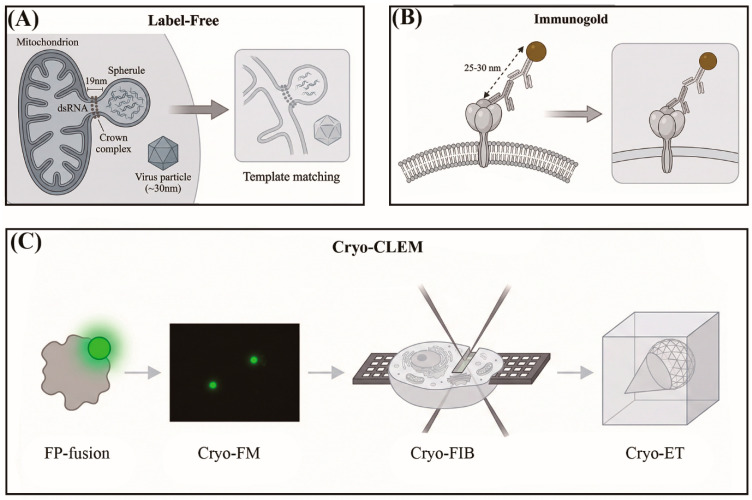
Labeling and tagging strategies for Cryo-ET of viral components. (**A**) Label-free identification using intrinsic morphology and template matching. (**B**) Native Cryo-immunogold labeling, illustrating ~25–30 nm spatial uncertainty. (**C**) Targeted Cryo-CLEM workflow utilizing fluorescent protein (FP) fusions.

The current reality is that, to our knowledge, no genetically encoded Cryo-ET tag has yet been validated on a viral assembly protein in a Cryo-ET experiment, and until systematic comparisons of tagged versus untagged viral lattices are performed, the structural neutrality of any tag on a viral assembly protein remains unverified. Existing applications are limited to fluorescent protein fusions used for Cryo-CLEM targeting, where the fluorescent signal guides data collection but the tag itself is not detected in the tomogram. Developing tags that are simultaneously small enough to be tolerated by viral assembly machinery and electron-dense enough to be detected in Cryo-ET represents perhaps the most important engineering challenge in the field. The principal labeling and tagging strategies discussed in this review are summarized in Figure 1.

## 5. Cryo-CLEM and Cryo-FIB-ET: Finding the Needle by Its Glow

Cryo-correlative light and electron microscopy (Cryo-CLEM) combines the molecular specificity of fluorescence microscopy with the structural detail of Cryo-ET [57,58]. The workflow involves fluorescence imaging of vitrified specimens to identify regions of interest, correlation of fluorescence coordinates onto the Cryo-EM grid, and Cryo-ET of the identified region. For thick cells, Cryo-FIB milling prepares electron-transparent lamellae (100–250 nm) through the region of interest while preserving vitrification [1,36]. This pipeline has become the standard approach for in situ structural virology in cells too thick for plunge-freezing alone. The relative strengths and limitations of each labeling strategy discussed in this review are summarized in Table 2.

Cryo-CLEM is particularly valuable for viral research because many events of greatest mechanistic interest are rare. A single nuclear pore complex engaging an HIV-1 capsid, a budding virion at the plasma membrane, or a viral RNA molecule entering a replication compartment each represents a low-probability event within any given Cryo-ET field of view. Fluorescence tagging reduces the search problem from a three-dimensional cellular volume to a defined coordinate set, dramatically increasing the yield of informative tomograms per unit beam time [57]. The foundations were established by Jun et al. [59], who correlated live-cell fluorescence of HIV-1 Gag with Cryo-ET of vitrified specimens, with subsequent advances in grid design, fiducial alignment, and FIB integration [60] enabling the high-throughput workflows that followed. More recent developments in correlative montage parallel array Cryo-tomography have further increased the throughput of targeted Cryo-ET acquisition [61]. Rodriguez et al. [62] combined time-resolved fluorescence imaging of HIV-1 capsid disassembly with end-point Cryo-ET of the same particles. Affinity capture of fluorescent HIV-1 particles onto Cryo-EM grids prior to vitrification and streamlined correlation protocols resolved distinct modes of capsid lattice stabilization corresponding to different disassembly kinetics under lenacapavir and inositol hexakisphosphate (IP6) treatment. This approach directly bridges dynamic single-molecule behavior with end-state structural information.

The correlative approach carries its own costs, which set the limits of what it can deliver. Correlation accuracy is finite: transferring a fluorescent coordinate from a cryo-light image onto the milled lamella typically leaves a residual uncertainty of tens to hundreds of nanometres, so fluorescence localizes a region rather than a single molecule, and the final assignment still rests on the tomographic density. Cryo-FIB milling removes most of the cellular volume to produce a 100–250 nm lamella, and a fluorescent target identified by light microscopy may simply lie outside the slab that survives milling, reducing the yield of usable events. Fluorophore photophysics at cryogenic temperature further constrain the workflow: photobleaching is suppressed, but emission is dim and the accessible photon budget is low, limiting the precision with which a signal can be centred. Cryo-CLEM therefore trades throughput and localization certainty for the ability to find rare events at all, and its yield depends on how faithfully the fluorescent tag reports the structural state of interest.

The most comprehensive application to date is the study of HIV-1 nuclear import by Hou et al. [25], in which mNeonGreen-labeled integrase (mNG-IN), previously validated by Mamede et al. [9], served as a fluorescence marker for intact cores in digitonin-permeabilized T cells. Cryo-CLEM identified cells with nuclear fluorescent signal, targeted Cryo-FIB milling generated lamellae through the nuclear envelope, and Cryo-ET produced tomograms of 1489 HIV-1 cores across four import stages. The data established that capsid elasticity and NPC adaptability are jointly required for translocation, building on the foundational observation by Zila et al. [24] that intact cone-shaped HIV-1 capsids traverse nuclear pores. A methodological caveat is that digitonin permeabilization removes cytosolic factors including cleavage and polyadenylation specificity factor 6 (CPSF6) and transportin-1 (TNPO1) that may regulate import in intact cells, and definitive confirmation will require capturing import in intact infected cells.

**Figure 2:**
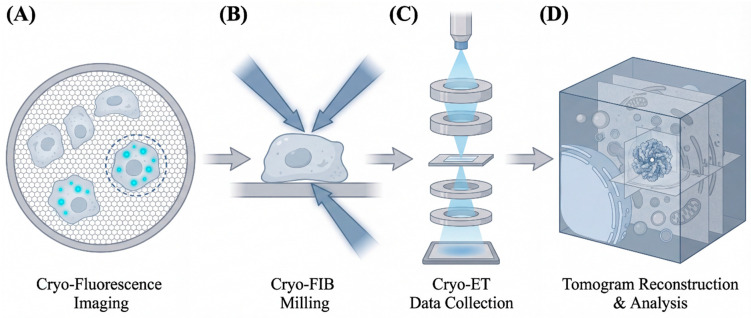
Schematic diagram of in situ structural analysis by Cryo-ET. (**A**) Cryo-fluorescence imaging of vitrified cells on an EM grid to identify regions of interest. (**B**) Cryo-FIB milling to produce an electron-transparent lamella. (**C**) Tilt-series acquisition across the lamella. (**D**) Tomographic reconstruction and subtomogram averaging of viral components in their native cellular context.

Beyond dissociated cell culture, Cryo-FIB-ET has been extended to intact multicellular tissue through Cryo-lamella lift-out. Klumpe et al. [26] applied this to Drosophila egg chambers to visualize Copia LTR retrotransposon virus-like particles (VLPs) in situ. Cryo-ET revealed size-heterogeneous cytosolic VLPs and size-homogeneous nuclear VLPs, with subtomogram averaging of nuclear capsids yielding a 7.7 Å structure without purification. No fluorescent tag or immunogold was required because nuclear VLPs are intrinsically size-distinguishable and abundant within germ cells. This contrasts directly with the HIV-1 system where Cryo-CLEM was indispensable, illustrating that event frequency and target abundance determine whether correlative targeting or label-free survey is the appropriate strategy. The overall Cryo-CLEM and Cryo-FIB-ET workflow for in situ structural analysis is summarized in Figure 2.

## 6. Conclusions and Future Perspectives

Cryo-ET has established itself as the method of choice for in situ structural analysis of viral components within their native cellular environment. The labeling strategies surveyed in this review span a continuum from non-perturbative label-free approaches to molecularly specific tagging methods, and the choice among them is determined by the intersection of viral biology with experimental accessibility. Label-free Cryo-ET is sufficient when viral assemblies present recognizable symmetry or distinctive morphology. Native Cryo-immunogold resolves a structurally ambiguous density when the epitope is accessible. Cryo-CLEM provides the targeting precision required when the event of interest is rare within the cellular volume.

Before turning to what remains unsolved, it is worth stating plainly what in situ Cryo-ET has already delivered for virology. Within the past five years it has resolved the 12-fold protein A crown that gates the FHV replication spherule [11,12], the approximately 3 MDa nsp3–nsp4 pore spanning the coronavirus double-membrane vesicle [13,14], the nsP1 capping ring of the alphavirus replication organelle [17,18], the progressive condensation of the Ebola nucleocapsid within infected cells [19,20], the passage of an intact HIV-1 capsid through the nuclear pore [24], and the assembly of influenza vRNP complexes at recycling endosomes [21], each in the native cellular context and, in most cases, without any exogenous label. These are not incremental refinements of purified structures; they are direct observations of viral machinery caught in the act. The labeling strategies surveyed here are the means by which the field will extend this catalogue to the components that remain invisible.

The contribution of this review is to organize these strategies around viral component type and life-cycle context rather than technique alone, providing a practical framework for matching a labeling approach to a specific structural question. The central conclusion that emerges is that genetic and synthetic Cryo-ET tags, although advancing rapidly for cellular protein applications, have not yet been demonstrated on a viral assembly protein. To our knowledge, no study has reported a side-by-side Cryo-ET comparison of a tagged and an untagged viral lattice, which is the evidence that would be required to establish that a tag is structurally neutral in this context. This is not a matter of incremental optimization. The assembly constraints inherent to viral systems make tagging a fundamentally harder problem than tagging cytoskeletal or signaling proteins, and closing this gap defines the most consequential engineering challenge in the field.

Several challenges shape the frontier, and their relative weight depends on the virological question at hand. Chemical identity remains unresolved for viral nucleic acids: current approaches detect bulk electron density consistent with dsRNA but cannot distinguish genome from replication intermediate, and no strategy yet identifies single viral RNA molecules in situ. Multiplexed labeling remains limited to two-component discrimination, far short of what fluorescence microscopy achieves routinely. Throughput remains low relative to the rarity of many life-cycle events, and temporal resolution remains inherent to any snapshot technique. Closing the tag gap will require new engineering rather than adaptation of existing tools. Split-system architectures that assemble an electron-dense reporter only at the site of interest could reduce steric burden on individual fusion sites, miniaturized metalloprotein tags could bring tag dimensions below the threshold that disrupts viral oligomerization, and computational prediction of tag-tolerant insertion sites could expand viable fusion positions beyond default termini. Realizing these directions, together with advances in AI-assisted detection and biosafety-compatible workflows for high-containment pathogens, will extend the experimental toolkit for in situ viral structural biology toward connecting molecular mechanism to cellular function across the viral life cycle.

## Figures and Tables

**Table 1:** Landmark in situ Cryo-ET discoveries of viral structures and the labeling strategy each required.

Virus/System	Structure Revealed In Situ	Labeling Strategy
Flock House virus (FHV)	Mitochondrial spherule interior; 12-fold symmetric protein A crown at the spherule neck (8.5 Å) [11,12]	Label-free; native Cryo-immunogold confirmation of replicase identity
SARS-CoV-2	12 + 12 nsp3–nsp4 pore in four stacked hexameric rings (~3 MDa, pseudo-12-fold) spanning the double-membrane vesicle [13,14]	Label-free (lumenal target; antibody-inaccessible)
SARS-CoV-2	Spike conformational states and three-hinge stalk flexibility on intact virions [15,16]	Label-free
Chikungunya virus	nsP1 dodecameric ring capping plasma-membrane spherules [17,18]	Label-free
Ebola virus	Progressive nucleocapsid condensation intermediates in infected cells [19,20]	Label-free (Cryo-FIB-ET)
Influenza A virus	vRNP clustering at Rab11a-positive recycling endosomes [21]	Label-free (Cryo-FIB-ET)
Human papillomavirus 16	Endolysosomal trafficking intermediates challenging the lysosomal disassembly model [22]	Label-free
HIV-1	Cone-shaped capsid traversing an intact nuclear pore complex [23,24]	Label-free
HIV-1	1489 cores across four nuclear-import stages; capsid elasticity and NPC adaptability [25]	Cryo-CLEM (mNG-IN) + Cryo-FIB-ET
Copia LTR retrotransposon	Nuclear VLP capsid at 7.7 Å in intact Drosophila egg chambers [26]	Label-free + Cryo-lamella lift-out

**Table 2:** Comparison of Labeling and Tagging Strategies for Cryo-ET of Viral Components.

Strategy	Size (nm)	Target Accessibility	Primary Limitation
Label-Free [1,27]	N/A	All compartments	Requires repetitive symmetry Monomeric proteins unidentifiable
Native Cryo-Immunogold [11,40]	25–30 †	Surface or mild permeabilization	Spatial uncertainty ~25–30 nm Intracellular targets largely inaccessible
FerriTag [46]	10–12	Intracellular	Requires rapalog induction; rapamycin inhibits mTOR, confounding viral processes
GEM/Encapsulin [48]	~25	Intracellular	Too large for capsid subunit fusion; steric clash with assembly interfaces
DNA-Origami SPOT [53]	30–100	Extracellular only	Cannot access intracellular viral components
Fluorescent Protein (Cryo-CLEM) [25,59]	3–4	Intracellular	No direct Cryo-ET contrast Requires correlative workflow

† Total localization uncertainty (epitope-to-gold center), including primary/secondary antibodies and the 5–15 nm gold particle.

## Data Availability

No new data were created or analyzed in this study. Data sharing is not applicable to this article.
